# Supplementary material for: Polo-like kinase-dependent phosphorylation of the synaptonemal complex protein SYP-4 regulates double-strand break formation through a negative feedback loop
Source: eLife. 2017 Mar 27;6:e23437. doi: 10.7554/eLife.23437 (PMC5423773; doi:10.7554/eLife.23437)
Supplement: Supplementary file 1. — The ‘Eggs Laid’ column indicates the average number of eggs laid (including both non-hatched and hatched embryos) per P0 hermaphrodite ± standard deviation. % Embryonic lethality was calculated by dividing the number of non-hatched embryos by the total number of hatched and non-hatched embryos laid. % Males was calculated by dividing the total number of males observed by the total number of hatched (viable) progeny scored. N = total number of P0 worms for which entire broods were scored. *p<0.0001 (Two-tailed Mann-Whitney test, 95% C.I.). DOI: http://dx.doi.org/10.7554/eLife.23437.025 [file elife-23437-supp1.doc]

| **GENOTYPE** | **EGGS**  **LAID** | **% EMBRYONIC LETHALITY** | **% MALES** | **N** |
| --- | --- | --- | --- | --- |
| Wild type | 261±29 | 0 | 0 | 15 |
| *syp-4(S269A)* | 167.9±27* | 6.4 | 0.3 | 15 |
| *syp-4(S269D)* | 207±29* | 7.9 | 1 | 15 |
