# Supplementary material for: Polo-like kinase-dependent phosphorylation of the synaptonemal complex protein SYP-4 regulates double-strand break formation through a negative feedback loop
Source: eLife. 2017 Mar 27;6:e23437. doi: 10.7554/eLife.23437 (PMC5423773; doi:10.7554/eLife.23437)
Supplement: Supplementary file 2. — Dunn’s multiple comparisons test between groups was applied to assess statistical significance of GFP::SYP-3 fluorescence recovery data presented in Figure 6B. LZ indicates leptotene/zygotene stage nuclei and MP indicates mid-pachytene stage nuclei. DOI: http://dx.doi.org/10.7554/eLife.23437.026 [file elife-23437-supp2.docx]

| Dunn's multiple comparisons test | Mean rank diff. | Significant? | Summary | Adjusted P Value |
| --- | --- | --- | --- | --- |
| LZ_WT vs. MP_WT | -49.32 | Yes | **** | <0.0001 |
| LZ_WT vs. LZ_*syp-4(S269A)* | 22.23 | No | ns | 0.5467 |
| LZ_WT vs. LZ_*syp-4(S269D)* | 54.92 | Yes | **** | <0.0001 |
| LZ_*syp-4(S269A)* vs. LZ_*syp-4(S269D)* | -32.7 | No | ns | 0.0670 |
| MP_WT vs. MP_*syp-4(S269A)* | -57.05 | Yes | **** | <0.0001 |
| MP_WT vs. MP_*syp-4(S269D)* | 24.39 | No | ns | 0.6209 |
| MP_WT vs. LZ_*syp-4(S269A)* | -27.1 | No | ns | 0.2770 |
| MP_WT vs. LZ_*syp-4(S269D)* | 5.6 | No | ns | >0.9999 |
| MP_*syp-4(S269A)* vs. MP_*syp-4(S269D)* | -81.44 | Yes | **** | <0.0001 |
| MP_*syp-4(S269A)* vs. LZ_WT | 7.729 | No | ns | >0.9999 |
| MP_*syp-4(S269A)* vs. LZ_*syp-4(S269D)* | 62.65 | Yes | **** | <0.0001 |
| MP_*syp-4(S269A)* vs. LZ_*syp-4(S269A)* | 29.95 | No | ns | 0.1379 |
| MP_*syp-4(S269D)* vs. LZ_WT | -73.71 | Yes | **** | <0.0001 |
| MP_*syp-4(S269D)* vs. LZ_*syp-4(S269D)* | -18.79 | No | ns | >0.9999 |
| MP_*syp-4(S269D)* vs. LZ_*syp-4(S269A)* | -51.48 | Yes | *** | 0.0002 |
